# Genomic diversity and antimicrobial resistance of Staphylococcus aureus in Saudi Arabia: a nationwide study using whole-genome sequencing

**DOI:** 10.1099/mgen.0.001540

**Published:** 2025-11-12

**Authors:** Mohammed S. Alarawi, Musaad Altammami, Mohammed Abutarboush, Maxat Kulmanov, Dalal M. Alkuraithy, Senay Kafkas, Robert Radley, Marwa Abdelhakim, Hind Aldakhil, Reema A. Bawazeer, Mohammed A. Alolayan, Basel M. Alnafjan, Abdulaziz A. Huraysi, Amani Almaabadi, Bandar A. Suliman, Areej G. Aljohani, Hassan A. Hemeg, Mohammed S. Almogbel, Meshari Alazmi, Abdulrahman S. Bazaid, Turki S. Abujamel, Anwar M. Hashem, Ibrahim A. Al-Zahrani, Mohammed S. Abdoh, Haya I. Hobani, Rakan F. Felemban, Wafaa A. Alhazmi, Pei-Ying Hong, Majed F. Alghoribi, Sameera Aljohani, Hanan Balkhy, Abdulrahman Alswaji, Maha Alzayer, Bassam Alalwan, Mai M. Kaaki, Sharif M. Hala, Omniya Ahmad Fallatah, Wesam Bahitham, Samer Zakri, Mohammad A. Alshehri, Nader Kameli, Abdullah Algaissi, Edrous Alamer, Abdulaziz Alhazmi, Amjad A. Shajri, Majid Ahmed Darraj, Bandar Kameli, O. O. Sufyani, Badreldin S. Rahama, Abrar A. Bakr, Fahad M. Alhoshani, Azzam A. Alquait, Ali M. Somily, Ahmed M. Albarrag, Lamia Alosaimi, Sumayh A. Aldakeel, Fayez S. Bahwerth, Mushtaq A. Khan, Tamir T. Abdelrahman, Séamus Fanning, Essam A. Tawfik, Essam J. Alyamani, Takashi Gojobori, Satoru Miyazaki, Mohammed B. Al-Fageeh, Robert Hoehndorf

**Affiliations:** 1 Biological and Environmental Sciences and Engineering (BESE) Division, King Abdullah University of Science and Technology, 4700 KAUST, Thuwal, Saudi Arabia; 2 Computational Bioscience Research Center, King Abdullah University of Science and Technology, KAUST, Thuwal, Saudi Arabia; 3 Computer, Electrical and Mathematical Sciences and Engineering (CEMSE) Division, King Abdullah University of Science and Technology, 4700 KAUST, Thuwal, Saudi Arabia; 4 Wellness and Preventive Medicine Institute, Health Sector, King Abdulaziz City for Science and Technology (KACST), Riyadh, Saudi Arabia; 5 SDAIA-KAUST Center of Excellence in Data Science and Artificial Intelligence, King Abdullah University of Science and Technology, KAUST, Thuwal, Saudi Arabia; 6 KAUST Center of Excellence for Smart Health (KCSH), King Abdullah University of Science and Technology, 4700 KAUST, Thuwal 23955, Saudi Arabia; 7 KAUST Center of Excellence for Generative AI, King Abdullah University of Science and Technology, 4700 KAUST, Thuwal 23955, Saudi Arabia; 8 Department of Biological Sciences, College of Science, University of Jeddah, Jeddah, Saudi Arabia; 9 Infectious Diseases Research Department, King Abdullah International Medical Research Centre(KAIMRC), Riyadh, Saudi Arabia; 10 King Saud bin Abdulaziz University for Health Sciences, Riyadh, Saudi Arabia; 11 College of Applied Medical Sciences, Taibah University, Madina, Saudi Arabia; 12 Department of Medical Laboratory Technology, College of Applied Sciences, Taibah University, Madina, Saudi Arabia; 13 BndrGene Medical Lab, Madina, Saudi Arabia; 14 Department of Medical Laboratory Sciences, College of Applied Medical Sciences, University of Hail, Hail, Saudi Arabia; 15 College of Computer Science and Engineering, University of Hail, Hail 81411, Saudi Arabia; 16 Department of Medical Laboratory Sciences, Faculty of Applied Medical Sciences, King Abdulaziz University, Jeddah 21589, Saudi Arabia; 17 EcoHealth Unit, King Fahd Medical Research Center, King Abdulaziz University, Jeddah, Saudi Arabia; 18 Vaccines and Immunotherapy Unit, King Fahd Medical Research Center, King Abdulaziz University, Jeddah 21589, Saudi Arabia; 19 Department of Clinical Microbiology and Immunology, Faculty of Medicine, King Abdulaziz University, Jeddah, Saudi Arabia; 20 Epidemiology Department, Public Health Administration, King Abdullah Medical Complex, Jeddah 23816, Saudi Arabia; 21 Alnoor Specialist Hospital, Ministry of Health, Makkah, Saudi Arabia; 22 Environmental Science and Engineering Program, Biological and Environmental Science and Engineering (BESE) Division, King Abdullah University of Science and Technology, Thuwal, Saudi Arabia; 23 Department of Pathology and Laboratory Medicine, King Abdulaziz Medical City (KAMC), Riyadh, Saudi Arabia; 24 King Abdullah International Medical Research Centre (KAIMRC), Riyadh, Saudi Arabia; 25 World Health Organization, Geneva, Switzerland; 26 Medical Laboratory, King Abdulaziz Medical City (KAMC), Ministry of National Guard Health Affairs, Jeddah, Saudi Arabia; 27 Ministry of National Guard Health Affairs, Riyadh, Saudi Arabia; 28 Biothreat Response Department, Public Health Laboratory, The Saudi Public Health Authority, Riyadh, Saudi Arabia; 29 King Abdullah International Medical Research Center-WR, King Saud bin Abdulaziz University for Health Sciences, Ministry of National Guard Health Affairs, Riyadh 11426, Saudi Arabia; 30 Ministry of National Guard Health Affairs, Jeddah, Saudi Arabia; 31 King Abdullah International Medical Research Center, Jeddah, Saudi Arabia; 32 King Saud bin Abdulaziz University for Health Sciences, Jeddah, Saudi Arabia; 33 King AbdulAziz University Hospital, Jeddah, Saudi Arabia; 34 Department of Medical Laboratory Technology, College of Nursing and Health Sciences, Jazan University, Jazan, Saudi Arabia; 35 Emerging and Endemic Infectious Diseases Research Unit, Health Research Center, Jazan University, Jazan, 45142, Saudi Arabia; 36 Department of Basic Medical Sciences, Faculty of Medicine, Jazan University, Jazan, Saudi Arabia; 37 Department of Medical Laboratories Technology, College of Applied Medical Sciences, Jazan University, Jazan, Saudi Arabia; 38 Department of Medicine, Faculty of Medicine, Jazan University, Jazan, Saudi Arabia; 39 Regional Laboratory & Central Blood Bank Jazan Health, Jazan, Saudi Arabia; 40 Saudi Public Health Authority, Vector-Borne Diseases Laboratory, Jazan 45142, Saudi Arabia; 41 Advanced Diagnostics and Therapeutics Institute, Health Sector King Abdulaziz City for Science and Technology (KACST), Riyadh, Saudi Arabia; 42 Department of Pathology, College of Medicine, King Saud University and King Saud University Medical City, Riyadh, Saudi Arabia; 43 The National Centre for Genomic Technology (NCGT), Life Science and Environment Research Institute, King Abdulaziz City for Science and Technology (KACST), Riyadh, Saudi Arabia; 44 Medical Microbiology Laboratory, Hera General Hospital, Makkah Healthcare Cluster, Makkah, Saudi Arabia; 45 Department of Medical Microbiology and Immunology, College of Medicine and Health Sciences, United Arab Emirates University, Al Ain, UAE; 46 King Faisal Specialist Hospital & Research Centre, Madina, Saudi Arabia; 47 UCD-Centre for Food Safety, University College Dublin, Belfield, Dublin D04 N2E5, Ireland; 48 Institute for Global Food Security (IGFS), The Queen's University of Belfast, Belfast BT95DL, Northern Ireland, UK; 49 Faculty of Pharmaceutical Sciences, Department of Pharmacy, Tokyo University of Science, Noda, Chiba, Japan

**Keywords:** antimicrobial resistance, genomic surveillance, mass gatherings, methicillin-resistant *Staphylococcus aureus* (MRSA), phylogenomics, Saudi Arabia

## Abstract

Methicillin-resistant *Staphylococcus aureus* (MRSA) surveillance in regions with mass gatherings presents unique challenges for public health systems. Saudi Arabia, hosting millions of pilgrims annually, provides a distinctive setting for studying how human mobility shapes bacterial populations, yet comprehensive genomic surveillance data from this region remain limited. Here, we present an integrated analysis of *S. aureus* isolates collected across seven Saudi Arabian regions, combining whole-genome sequencing with extensive antimicrobial susceptibility testing and standardized metadata following findability, accessibility, interoperability and reusability data principles. Our analysis revealed striking differences between pilgrimage and non-pilgrimage cities. Pilgrimage cities showed significantly higher genetic diversity and antimicrobial resistance rates, harbouring numerous international strains, including recognized clones from diverse geographic origins. Reported lineage dynamics are changing, expanding toward community clones. While genomic prediction of antimicrobial resistance showed high accuracy for some antibiotics, particularly beta-lactams, with varying performance for others, it highlights the necessity for phenotypic testing in clinical settings. Our findings demonstrate how mass gatherings drive bacterial population structures and emphasize the importance of integrated surveillance approaches in regions with significant global connectivity and travel.

**Impact Statement**

We generated genomic sequences from 686 *Staphylococcus aureus* isolates collected across seven regions of Saudi Arabia and paired these with antimicrobial susceptibility phenotyping data. This represents a large-scale, nationally representative genomic dataset for *S. aureus* from Saudi Arabia, revealing how mass gatherings drive bacterial population structures and resistance patterns. We made all data publicly available following findability, accessibility, interoperability and reusability principles, providing both raw sequences and standardized metadata that enable integration with global surveillance efforts. By creating reproducible computational workflows and structured datasets linking genotypes to phenotypes, we provide an important resource for tracking pathogen evolution in a region with substantial global connectivity and for developing machine learning approaches to predict antimicrobial resistance.

## Data Summary

Sequencing data is available on the Sequence Read Archive as a BioProject under accession number PRJEB59751. The phenotypes for all samples are available on Zenodo under DOI 10.5281/zenodo.14250852, https://zenodo.org/records/14250852 [45].

## Introduction

*Staphylococcus aureus*, a Gram-positive bacterium characterized by its grape-like clustering morphology, is both a common human and animal commensal organism and a significant pathogen that has emerged as a leading cause of hospital-acquired infections. Notably, methicillin-resistant *S. aureus* (MRSA) accounts for ~25–50% of these infections. MRSA emerged shortly after methicillin’s introduction in 1958, following the acquisition of a mobile genetic element known as the staphylococcal chromosomal cassette *mec* (SCC*mec*) [1,3]. This cassette harbours the *mec*A gene, which encodes PBP2A, an alternative penicillin-binding protein involved in cell-wall synthesis. The modified protein exhibits reduced affinity to *β*-lactam antibiotics, conferring broad-spectrum resistance to this antibiotic class [4].

The global impact of MRSA has expanded significantly beyond healthcare settings, establishing distinct reservoirs within communities and livestock populations [5,7]. Understanding the epidemiological distribution of MRSA lineages and their corresponding resistance phenotypes across communities, regions and countries has become increasingly crucial from a public health perspective. This knowledge directly informs the development of targeted mitigation strategies, encompassing evidence-based policies for antibiotic stewardship, systematic screening protocols and comprehensive livestock management practice [8,10]. The clinical significance of MRSA is particularly evident in its role as a major pathogen in post-surgical complications, community-onset infections and foodborne illness outbreaks [6,11].

Saudi Arabia presents a unique context for studying MRSA transmission and resistance patterns. As the largest country on the Arabian Peninsula by both area and population, it holds profound religious significance as the custodian of Islam’s two holiest sites. This unique position draws millions of Muslim pilgrims annually to the Kingdom. Mass gatherings increase chances for microbial transmission dynamics and the introduction of novel clones and antimicrobial resistance patterns. Large cities are hypothesized to function as entry points and subsequent dissemination of imported clones due to population density and connectivity. Consequently, international clones are predicted to emerge initially in these entry points before spreading to other regions within Saudi Arabia. Smaller, less connected cities are expected to display low presence of these clones and lower diversity [11,12]. The epidemiology of MRSA in Saudi Arabia has been documented through various investigations [11,13,15], although most studies have been limited to individual healthcare facilities or specific geographical regions. These investigations range from comprehensive phenotypic characterization of MRSA isolates’ antimicrobial susceptibility profiles [16,18] to more detailed molecular analyses of strain characteristics [13].

Despite extensive clinical research on MRSA in Saudi Arabia, a significant gap remains in comprehensive datasets that integrate both genotypic and phenotypic information across multiple geographical regions and research areas, particularly concerning the ‘One Health’ approach [6]. One critical component to address this gap is the use of findable, accessible, interoperable and reusable (FAIR) data principles [19]. Implementation of FAIR increases the value of accumulated data by enabling easier reuse and interoperability. Interoperable data are valuable because they can be combined with other datasets and aid in elucidating molecular mechanisms of antimicrobial resistance and identifying novel therapeutic targets, in addition to transmission burden. Moreover, combined genotype–phenotype datasets enable the development of machine learning approaches for predicting drug resistance patterns and pathogenicity profiles, while also informing the design of targeted studies aligned with the One Health approach [20].

To address this knowledge gap, we established a collection of 686 *S*. *aureus* isolates from diverse regions across Saudi Arabia, encompassing clinical specimens, community screening samples and wastewater isolates. We conducted whole-genome sequencing on the isolates and performed extensive drug resistance phenotyping screening, creating a novel geospatial genotype–phenotype dataset. Our analysis reveals high genetic diversity, including several previously unidentified sequence types distributed across different regions. These novel sequence types exhibit elevated patterns of drug resistance. Significantly, we observed higher genetic diversity and increased levels of drug resistance in cities associated with mass gatherings (Jeddah, Makkah and Madina), suggesting that mass gatherings may serve as drivers of antimicrobial resistance evolution and transmission of *S. aureus* clones. These findings provide crucial insights for developing targeted intervention strategies and underscore the importance of genomic surveillance in regions experiencing regular mass gatherings.

## Methods

### Sample selection and sourcing

Our study includes a total of 686 *S*. *aureus* isolates collected from seven distinct regions across Saudi Arabia. The majority of samples were obtained from tertiary care hospitals, with the Western region contributing the largest proportion: Jeddah (275 isolates), Madina (102 isolates) and Makkah (36 isolates). Additional regional collections include Riyadh in the Central region (158 isolates), Hail in the North (64 isolates), Al Hasaa in the East (33 isolates) and Jazan in the South (18 isolates). To broaden the scope of our surveillance, we supplemented the hospital-sourced isolates with 60 samples collected through a community screening of healthy individuals in Jeddah city and 5 environmental wastewater samples from the same region (all of which are included in the 275 isolates assigned to ‘Jeddah’). A detailed breakdown of sample distribution is provided in Table S1, available in the online Supplementary Material. Sample selection within each hospital followed a random sampling approach, and we included both MRSA and methicillin-susceptible *S. aureus* (MSSA) isolates to ensure comprehensive representation of circulating strains.

### Bacterial identification and susceptibility testing

All isolates were initially enriched on tryptic soy agar supplemented with 5% (w/v) NaCl (Sigma-Aldrich, Germany) to selectively culture *S. aureus*. Single colonies were used for subsequent bacterial identification, susceptibility testing and glycerol stock archival. Throughout the identification process, the *S. aureus* ATCC 29213 (NCTC 12973) strain served as an internal reference control. Phenotypic drug resistance profiling was performed using the VITEK 2 System with GP ID cards for identification and AST-P580 cards for antimicrobial susceptibility testing (bioMérieux, France). Table S2 shows the antimicrobial agents and their concentrations on the AST-P580 card. All test results were digitally recorded and stored for analysis.

### DNA isolation

Genomic DNA isolation was performed using single colonies obtained either directly from Petri dishes or from enriched broth cultures following the identification. For each sample, 2 ml of the overnight culture was pelleted for DNA extraction. Concurrently, we prepared archived samples by combining 500 µl molecular-grade glycerol (Thermo Fisher, USA) with 500 µl of the culture for future investigations. Genomic DNA was isolated using the automated KingFisher MagMax DNA isolation kit protocol (Thermo Fisher, USA). DNA quality and quantity were assessed using both a NanoDrop instrument (Thermo Fisher, USA) and Qubit dsDNA BR Kit (Thermo Fisher, USA).

### Library preparation and DNA sequencing

Genomic DNA libraries were constructed using 100 ng of input DNA with the QIASeq FX DNA Library Kit (QIAGEN, Germany) following the manufacturer’s protocol. Library quality was assessed using an Agilent Bioanalyzer system (Agilent, USA). Whole-genome sequencing was performed on the Illumina NovaSeq 6000 platform (Illumina, USA) using SP flow cells, generating 150-base paired-end reads with average insert sizes ranging from 350 to 450 bp and 50–100× sequence depth.

### Genomic analysis

Initial quality control and preprocessing of raw sequencing reads were performed using TrimGalore v0.4.4 for adapter trimming [21]. Taxonomic identification and contamination were utilized with Kraken v2.0.8 beta [22] and Mash v2.3 [23]. Genomes were assembled using SKESA v2.4.0 [24] and called variants using Snippy v4.6.0 against the reference genome NC_007795.1. Contaminated genomes, or low-coverage ones, were excluded from the analysis using output from kraken, mash and quast [25].

Annotation of the assembled genomes utilized prokka v1.14.6 [26] and Roary for pangenome construction v3.13.0 [27]. Scoary 1.6.16 was used to perform a bacterial genome-wide association study (GWAS) for drug resistance phenotypes with Bonferroni-corrected *P*-value <0.05 [28]. A maximum likelihood phylogenetic tree was constructed from the core genome SNP alignment using IQ-TREE v1.6 [29] based on the core genome alignment. We aimed to resolve broad population structure and clonal complexes and removed recombination using Gubbins, which might influence branch length [30]. The resulting phylogenetic tree was visualized and annotated with metadata, including clonal complex, SCC*mec* type, geographic origin and key virulence markers, using the Interactive Tree Of Life (iTOL) [31]. Assignment of multilocus sequence types (MLST) was performed using ABRicate v1.0.1 with the MLST tool [32,33]. A minimum spanning tree was constructed using Phylovis v2 from core SNPs [34].

Multiple approaches were employed for antimicrobial resistance analysis. Drug resistance genes were identified using both the CARD v5.1.1 [35] and ResFinder v4.0 [36] databases via ABRicate v1.0.1 [32]. Additionally, DeepARG v1.0.2 [37], a deep learning approach, is used to enhance resistance gene detection. We compared the performance of the different methods by calculating three standard metrics: precision (P), recall (R) and the F1 score. (P) measures the proportion of true positives among all positive predictions [P=TP/(TP+FP)]; high precision indicates few false positives. (R) measures the proportion of true positives that were correctly identified [R=TP/(TP+FN)]; high recall indicates few false negatives. The F1 score is the harmonic mean of precision and recall [F1=2×(P×R)/(P+R)], providing a single balanced measure of a test’s accuracy. The best-performing tools are determined by the highest F1 score for each antibiotic. NA indicates that no predictions could be made by the tool for this antimicrobial agent. Virulence factors were identified using the virulence factor database [38] through ABRicate v1.0.1 [32].

Methicillin resistance mechanisms were characterized by screening for the *mecA* gene using the staphopia-Scc*mec* v1.0.0 typing tool [39]. The analysis was complemented using a local database containing whole cassette sequences (*mec*A, *mec*B and *mec*C), cassette chromosomal recombinase (*ccr*) genes and insertion sequences for each SCC*mec* type. Additional *mec*A cassette detection was performed using minimap2 v2.24-r1122 [40] with the local database. The SCC*mec* type assignments were determined based on cassette element arrangements following the workflow at https://github.com/cdnstp/SCCmec_CLA Finally, protein A gene polymorphism (*spa*) typing was performed using spaTyper v4.6.1 [41] with its associated database.

### Reproducibility and implementation of FAIR data principles

We implemented our entire analysis workflow using the common workflow language (CWL) [42] to ensure computational reproducibility. The two main analysis workflows are shown in Figs S1 and S2.

We executed all analyses on a local Arvados system [43], with complete workflow definitions and execution environments preserved. To promote FAIR, we have made all source code, workflow definitions and configuration files freely available at https://github.com/bio-ontology-research-group/mrsa-sequences. The repository includes detailed documentation of computational requirements, software versions and usage instructions. Additionally, raw sequencing data were deposited in the European Nucleotide Archive under accession number PRJEB59751. Furthermore, the metadata of our samples, as well as the measured antimicrobial resistance phenotypes using the Resource Description Framework (RDF) [44], are made available in a public repository [45] and on a GitHub repository. Fig. S3 describes the RDF data model.

## Results

### Genetic diversity and geographic distribution of *S. aureus* in Saudi Arabia

A total of 686 samples of *S. aureus* from multiple regions in Saudi Arabia were sequenced, following quality control validation and confirmation as *S. aureus* through whole-genome sequencing and phenotypic testing. The resulting high-quality draft genomes had a mean size of 2.85±0.05 Mbp and a mean G+C content of 32.82±0.05 mol%, consistent with known values for the species. The core genome alignment constructed from all isolates using Snippy-core spanned ~2.56 Mbp and a total of ~25,000 SNPs and average pairwise SNP difference (~22,500 SNPs), indicating a substantial genetic diversity across the dataset. The isolates consist of MRSA and MSSA strains, with MSSA proportions varying significantly by region: from 3.9% in Madina to 34.4% in Hail (Table 1).

**Table 1.** Summary of MRSA statistics across different geolocations, including total counts, unique clonal complexes (CCs), unique sequence types (STs), SCC*mec* type rate, Panton–Valentine leukocidin (PVL) rate and toxic shock syndrome toxin (TSST) rate

Geolocation	Total sample	Unique CC	Unique ST	SCC*mec* type rate%	PVL rate %	TSST rate %
Alhasa (east)	33	8	15	MSSA 6.1, IVa 45.5, IVc 12.1, V 30.3, VI 6.1	24.2	6
Hail (north)	64	9	19	MSSA 34.4, IIIa 3.1, IVa 28.1, IVc 4.7, V 23.4, VI 6.3	6.3	26
Jazan (south)	18	4	6	IVa 27.8, IVd 11.1, V 61.1	22.2	0
Jeddah (west)	275	9	43	MSSA 10.2, IVa 32.4, IVc/IVd 3.6, V 45.1, VI 2.9, VI/Vc/IVh/IIIa 0.4, undetermined 0.7	13.5	8
Madina (west)	102	9	26	MSSA 3.9, I 2.0, IVa 33.3, IVc 13.7, IVg 2.0, V 34.3, VI 6.9, Others 1.0	32.4	13
Makkah (west)	36	8	14	MSSA 13.9, IVa 38.9, IVc 2.8, V 41.7, undetermined 2.8	13.9	8
Riyadh (central)	158	9	29	MSSA 18.4, IVa 19.6, IVb 1.9, IVc 8.9, IVd 4.4, IVg/IVh 0.6, V 41.8, VI 3.2	19.0	7

**Fig. 1.**
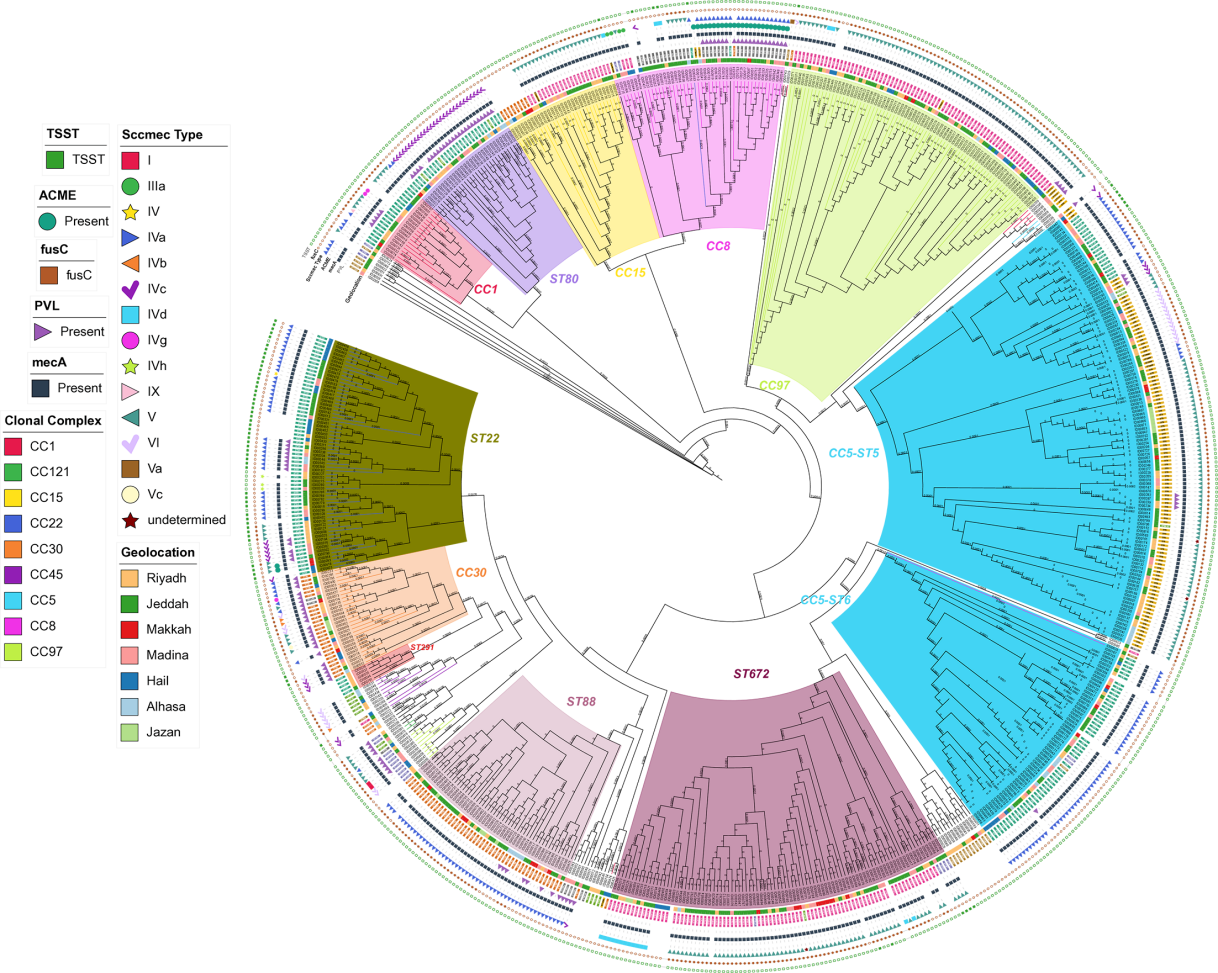
Phylogenomic structure and diversity of *S. aureus* across Saudi Arabia. Maximum likelihood tree constructed from core genome SNPs. Major CCs and grouping-related STs are highlighted (the dominant CC5, CC8, CC22 and ST672). Coloured rings display the associated SCC*mec* type and geographical region of origin, revealing the distribution patterns of major lineages. CC5 has two branches (ST5 and ST6) and the distinct clustering reflects national population structure influenced by regional and international factors. Key virulence/resistance markers (TSST, PVL, ACME and fusC) are indicated in the outermost tracks.

To understand the population structure and evolutionary relations of the isolates, we performed molecular typing and classification utilizing the PubMLST database [33], as this knowledge is crucial for tracking transmission patterns and implementing targeted infection control measures. The classification revealed nine major clonal complexes (CCs), with CC5 (26.38%), CC97 (12.39%) and CC22 (9.48%) being the most prevalent (Fig. S4). 31.92% of isolates belonged to previously unassigned clonal complexes. Our analysis also identified 14 novel sequence types (Table S3). Fig. 1 provides an overview of the molecular diversity and relatedness of the samples included in our study, in addition to genetic distance based on the core single-nucleotide variant minimum spanning tree (Fig. S5).

**Fig. 2.**
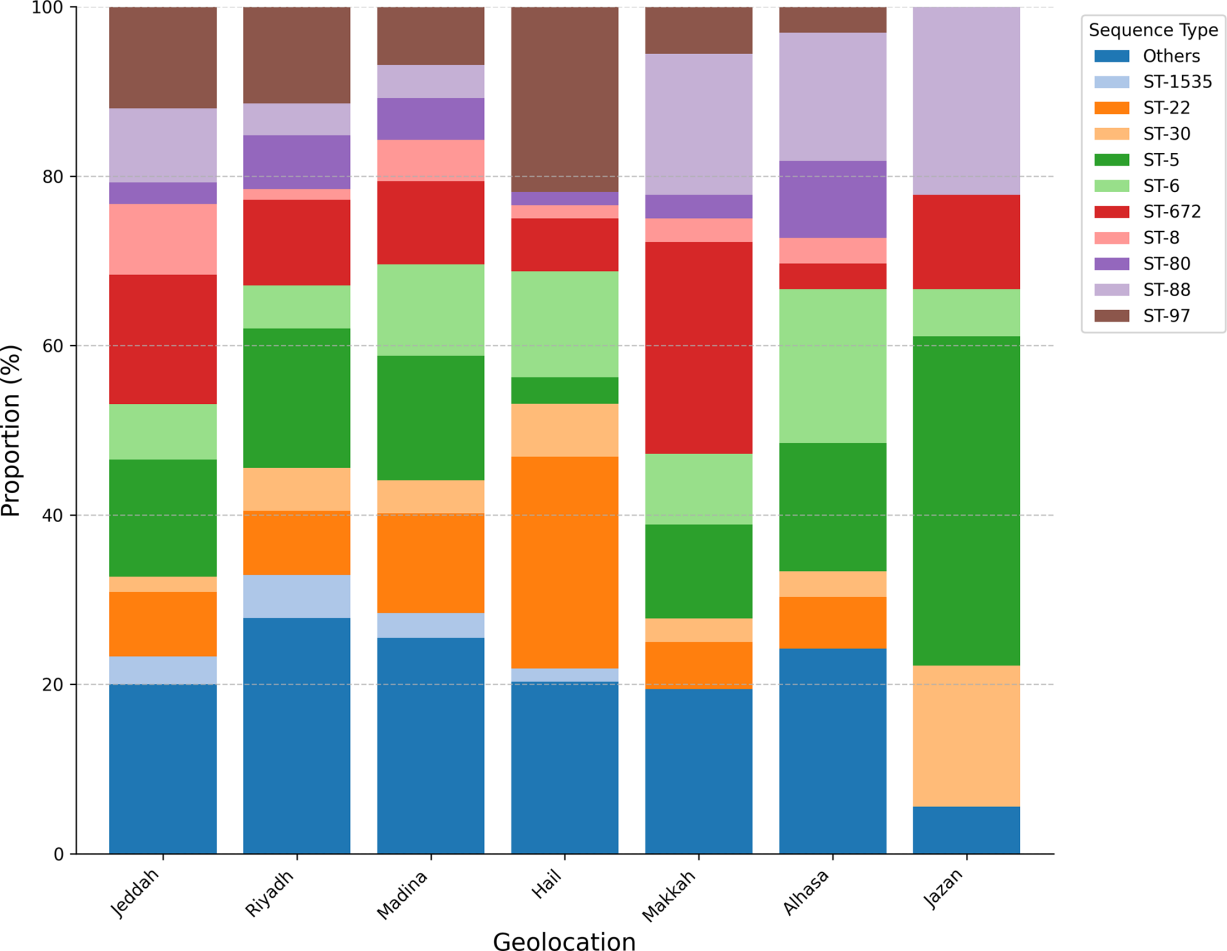
Stacked bar chart illustrating the relative proportion (%) of the top 10 most frequent *S. aureus* isolate ST per region from Saudi Arabia. The chart highlights the greater ST diversity observed in major pilgrimage-associated cities (Jeddah, Madina and Makkah) and other high-traffic locations like Riyadh, compared to regions like Jazan, which shows dominance by fewer STs (e.g. ST5 and ST30). Notable STs like ST5, ST97, ST672, ST8 and ST22 show varying prevalence across regions, indicating complex population structures influenced by both local factors and international travel patterns.

We observed distinct patterns of strain distribution aligned with pilgrimage routes in major cities. Cities along the Hajj and Umrah routes exhibited high genetic diversity: Jeddah (9 CCs and 43 STs), Madina (9 CCs and 26 STs) and Makkah (8 CCs and 14 STs), likely reflecting the international convergence of travellers. In contrast, regions with less international traffic showed more limited diversity, particularly Jazan (4 CCs and 6 STs). To quantitatively compare the diversity across regions while accounting for differences in sample size, we calculated Simpson’s index of diversity. The sequence type (ST) diversity index confirmed the high diversity in pilgrimage-associated cities like Madinah (0.934) and Jeddah (0.917) compared to the significantly lower diversity observed in Jazan (0.797) (Table S4). Analysis of the SCC*mec* revealed region-specific patterns, with SCC*mec*V ranging from 23.4% in Hail to 61.1% in Jazan and SCC*mec*IVa ranging from 19.6% in Riyadh to 45.5% in AlHasa (Table 1, Figs2^ 3^ and S6).

**Fig. 3.**
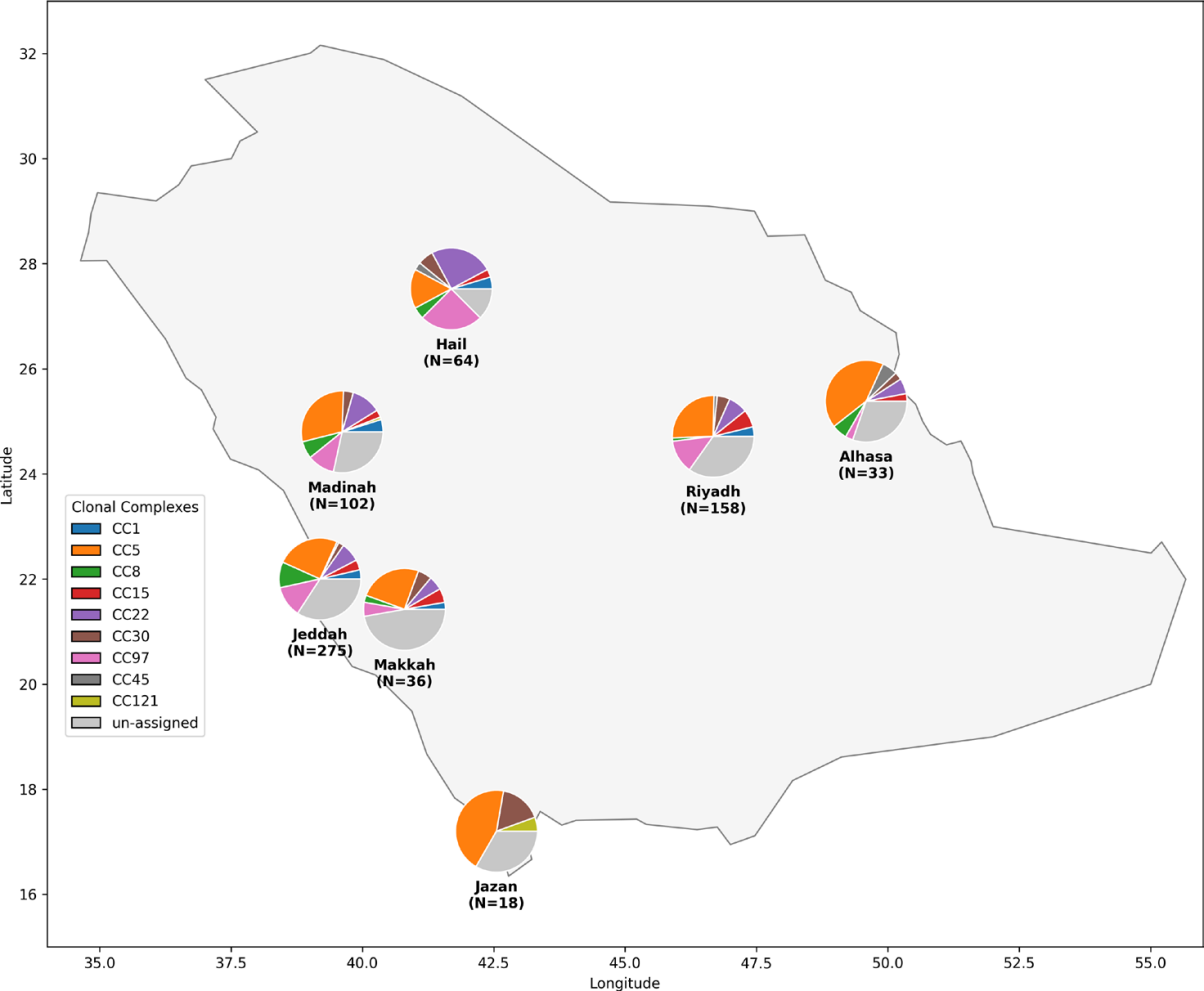
Analysis of *S. aureus* CCs in Saudi Arabia. The map displays the proportional distribution of major CCs among *S. aureus* isolates collected from multiple regions. The size of each pie chart slice corresponds to the percentage of isolates belonging to a specific CC. This distribution reveals a heterogeneous population structure, with some lineages, in particular CC5, demonstrating widespread prevalence, while others show more localized patterns. Isolates not assigned to a CC based on the PubMLST database are shown as ‘unassigned’.

Several internationally recognized strains were observed in pilgrimage cities. The Bengal Bay clone (CC1-ST772-PVL positive) [46] and CC30-ST1482 [47] were detected primarily in Makkah and Jeddah, regions that host large numbers of international pilgrims. The South Pacific Clone (CC30-ST30) was found across most cities [48].

### Phenotypic antimicrobial resistance

Antimicrobial susceptibility testing was performed on all isolates (Table 2). We observed the highest resistance rates for benzylpenicillin across all regions, ranging from 88.9% in Al Hasa to 100% in Makkah. Oxacillin resistance, mediated by *mec*A, showed considerable variation, from 60.3% in Hail to 96.6% in Madina. Fusidic acid emerged as the next most prevalent resistance, with particularly high rates in Riyadh (70.5%) and Jeddah (70.9%) but notably lower in Hail (35.3%). Fluoroquinolone resistance varied significantly by region, with levofloxacin resistance ranging from 14.7% in Hail to 45.7% in Madina, and similar patterns for moxifloxacin (13.4%–41.5%). Erythromycin resistance rates ranged from 19.4% in Al Hasa to 37.9% in Madina. We observed variable resistance to aminoglycosides, with tobramycin resistance ranging from 8.1% in Makkah to 32.5% in Jeddah, while gentamicin resistance showed similar patterns. Tetracycline resistance rates varied considerably, from 6.2% in Al Hasa to 26.5% in Jeddah.

**Table 2.** Antimicrobial resistance rates and Clinical and Laboratory Standards Institute (CLSI) clinical breakpoints for *S. aureus* The table shows the percentage (%) of isolates from each region classified as resistant based on minimum inhibitory concentration values.

Antimicrobial agent	CLSI breakpoint (µg ml^−1^) for resistance (R)	Riyadh (*n*=158)	Jeddah (*n*=275)	Makkah (*n*=36)	Madina (*n*=102)	Hail (*n*=64)	Alhasa (*n*=33)
Benzylpenicillin	≥0.25 (or *β*-lactamase POS)	98.3%	97.4%	100.0%	97.4%	95.6%	88.9%
Oxacillin	≥4	81.8%	90.6%	86.5%	96.6%	60.3%	93.9%
Gentamicin	≥16	18.2%	31.6%	0.0%	22.4%	20.6%	13.9%
Tobramycin	≥16	26.7%	32.5%	8.1%	27.8%	20.6%	13.9%
Levofloxacin/moxifloxacin	≥4 (Levo), ≥2 (Moxi)	27.3%/21.6%	23.9%/19.3%	40.5%/33.3%	45.7%/41.5%	14.7%/13.4%	25.0%/25.0%
Erythromycin	≥8	27.8%	25.6%	37.8%	37.9%	25.0%	19.4%
Clindamycin	≥4	19.3%	23.1%	18.9%	25.9%	20.6%	19.4%
Tetracycline	≥16	20.5%	26.5%	10.8%	20.9%	7.4%	6.2%
Fusidic acid	(No CLSI breakpoint)	70.5%	70.9%	62.2%	60.9%	35.3%	46.9%
Rifampicin	≥4	0.0%	0.9%	0.0%	1.7%	0.0%	0.0%
Nitrofurantoin	≥128	0.0%	0.9%	0.0%	1.7%	0.0%	0.0%
Vancomycin	≥16	0.0%	0.0%	0.0%	0.0%	0.0%	0.0%
Linezolid	≥8	0.0%	0.0%	0.0%	0.0%	0.0%	0.0%
Teicoplanin	≥32	0.0%	0.0%	0.0%	0.0%	0.0%	0.0%
Tigecycline	(No CLSI breakpoint)	0.0%	0.0%	0.0%	0.0%	0.0%	0.0%

Several antibiotics maintained complete efficacy across all regions. We observed no resistance to linezolid, tigecycline or teicoplanin. Vancomycin, often considered a last-resort antibiotic [49], showed nearly complete effectiveness, with only a single borderline case identified in a sample from Madina with updated minimum inhibitory concentration (MIC) break point. Similarly, nitrofurantoin and rifampicin maintained high effectiveness, with resistance rates not exceeding 1.7% in any region.

Comparison of overall differences in antimicrobial resistance was performed for locations associated with mass gatherings and other cities (Fig. S8). Pilgrimage-associated cities (Madina, Makkah and Jeddah) showed significantly higher overall resistance rates compared to other locations (33.9%) vs. 27.6%, *P*=0.047, one-tailed *t*-test).

### Genotypic identification of antimicrobial resistance

While whole-genome sequencing data identify resistance-associated genotypes, linking these directly to observed phenotypes remains a complex process. We compared phenotypic resistance results to genetic predictions using three approaches: CARD [35], ResFinder [36] and the deep learning tool DeepARG [37]. Genetic predictions showed varying levels of concordance with phenotypic resistance across different antibiotic classes (Table 3).

**Table 3.** Concordance of antimicrobial resistance predictions with the observed resistance patterns using three tools (CARD database, ResFinder 4.0 and DeepARG)

	CARD			ResFinder			DeepARG			
**Antimicrobial agent**	**Precision**	**Recall**	**F1 score**	**Precision**	**Recall**	**F1 score**	**Precision**	**Recall**	**F1 score**	**Best-performing tool**
Benzylpenicillin	0.983	0.980	0.982	0.994	0.572	0.726	0.983	0.980	0.982	CARD, DeepARG
Cefoxitin	1.000	0.004	0.007	0.984	0.950	0.967	n/a	n/a	n/a	ResFinder
Oxacillin	0.858	0.976	0.913	n/a	n/a	n/a	0.858	0.976	0.913	CARD, DeepARG
Fusidic acid	0.937	0.925	0.931	0.940	0.845	0.890	1.000	0.005	0.010	CARD, ResFinder
Erythromycin	0.283	1.000	0.441	0.776	0.889	0.828	0.584	0.895	0.707	ResFinder
Clindamycin	0.446	0.861	0.588	0.704	0.826	0.760	0.378	0.861	0.525	ResFinder
Tetracycline	0.167	0.962	0.285	0.853	0.771	0.810	0.491	0.800	0.609	ResFinder
Tobramycin	0.220	0.950	0.357	0.785	0.807	0.796	0.221	0.950	0.358	ResFinder
Gentamicin	0.172	0.937	0.291	0.752	0.793	0.772	0.172	0.937	0.291	ResFinder

Beta-lactam predictions demonstrated high accuracy, particularly for benzylpenicillin, where both CARD and DeepARG achieved identical high performance (precision 0.983, recall 0.980), while ResFinder showed higher precision (0.994) but lower recall (0.572). Oxacillin predictions showed strong agreement between CARD and DeepARG (precision 0.858, recall 0.976), while ResFinder did not provide predictions for this antibiotic. For fusidic acid, both CARD and ResFinder achieved accurate results (precision 0.937 and 0.940, respectively), while DeepARG could only make a few predictions.

However, we observed substantial discrepancies for other antibiotic classes. Aminoglycoside resistance predictions varied markedly across tools. For gentamicin and tobramycin, CARD and DeepARG showed low precision (0.172–0.221) but high recall (0.937), while ResFinder maintained more balanced performance (precision 0.752–0.785, recall 0.793–0.807). Similarly, predictions for macrolides and lincosamides showed moderate concordance; erythromycin and clindamycin predictions showed only low concordance with phenotypic observations.

To test for the presence of known and potentially novel mechanisms of antimicrobial resistance, we constructed a pangenome using Roary and visualized the pangenome using phandango (Fig. S9) [50] and performed a pangenome-wide association study using Scoary [28] (Table S5). The result of the bacterial GWAS identified known mechanisms of antimicrobial resistance. Genes such as *mecA* and *mecR1* were strongly associated with oxacillin and cefoxitin resistance. Similarly, aminoglycoside resistance was determined by *aacA-aphD* and *knt* for tobramycin; tetracycline resistance was shown to be mediated by efflux pump *tet(K)*; and the genes *ermC* and *msrA* were associated with resistance to macrolides and lincosamides (erythromycin and clindamycin).

However, non-canonical associations were present for other drug classes. For instance, fluoroquinolone resistance was associated with genes such as *hsdM* and *entC2*. Benzylpenicillin resistance was associated predominantly with *fcl_1*. Fusidic acid resistance showed associations primarily with transport-related genes (*natA*, *macB* and *dppB*).

### Virulence and pathogenicity

The prevalence and distribution of toxin and virulence factors indicate regional variation (see Fig. 1 and Table S6). Panton–Valentine Leukocidin (PVL), a cytotoxin often associated with community-associated MRSA, was detected in 17% of isolates overall. However, its prevalence showed marked regional differences, being highest in Madina (32.4%) and lowest in Hail (6.3%). Notably, PVL positivity was linked to specific lineages found predominantly in pilgrimage cities, including the internationally recognized CC1/ST772 (Bengal Bay clone).

Toxic shock syndrome toxin (TSST) was found in 10% of isolates and TSST rates showed an inverse geographic distribution pattern, ranging from 0% in Jazan to 26% in Hail, with intermediate rates in pilgrimage cities: Jeddah (8%), Makkah (8%) and Madina (13%). Western cities (Jeddah, Madina and Makkah) harboured the majority of novel ACME-positive isolates.

Core virulence factors displayed widespread but distinctive distribution patterns across our isolates. Adhesion factors showed varying prevalence: clumping factors (*clfA* and *clfB*) appeared in over 95% of isolates, while the collagen-binding protein (*cna*) occurred exclusively in CC5. The majority of isolates (>90%) carried immune evasion genes (*sak*, *sbi* and *scn*) and biofilm formation genes (*ica*ABCD cluster). Among toxin genes, *sea* dominated with 30% prevalence, followed by other enterotoxins (*seb*, *sec*, *sed* and *seh*).

## Discussion

### Impact of mass gatherings on MRSA diversity and resistance patterns

This study described the genetic epidemiology of *S. aureus* across Saudi Arabia, a country with over 32 million inhabitants, of which 41% are immigrants [51]. As of 2023, Saudi Arabia hosts over 2 million pilgrims for Hajj and 27 million pilgrims for Umrah annually [52]. While previous work has investigated the effect of mass gatherings and pilgrimage on antimicrobial resistance in Saudi Arabia [53], our integrated genotype–phenotype dataset reveals several distinct patterns associated with mass gathering activities.

The impact of mass gatherings on MRSA epidemiology is particularly evident in cities along the Hajj and Umrah routes. Jeddah, Makkah and Madina showed significantly higher genetic diversity and unique strain patterns compared to other regions, with Jeddah, the main entry point for pilgrims, exhibiting the highest number of novel sequence types. This pattern aligns with observations from other Gulf regions, where mass gatherings coincide with diverse strain distributions [48,54,56]. This diversity also includes a prevalence of internationally recognized strains, such as the PVL-positive Bengal Bay clone (CC1-ST772) and the South Pacific clone (CC30-ST30), which are found in multiple cities, including entry points, suggesting a link between pilgrimage routes and the importation of globally circulating lineages [57].

These findings suggest that mass gatherings influence not only genetic diversity but also antimicrobial resistance patterns. Pilgrimage cities showed significantly higher overall resistance rates compared to other locations, suggesting these sites may serve as hotspots for resistance transmission. This is particularly evident in the distribution of SCC*mec* types, which suggests a shift from hospital-associated to community-associated MRSA, reflecting global trends [58,59]. The concentrated presence of ACME-positive isolates in western pilgrimage cities further supports the role of international travel in strain dissemination.

The successful persistence of these strains in mass gathering-associated regions appears to be facilitated by multiple factors. Genomic analysis revealed a high presence of key genes associated with host colonization and immune evasion. Specifically, genes encoding for biofilm formation, such as the *ica* operon (*ica*A, *ica*B, *ica*C and *ica*D), were detected in over 90% of all isolates, indicating a strong intrinsic capacity for surface attachment and biofilm formation. Similarly, a core set of immune evasion genes was highly conserved. Genes for staphylokinase (*sak*), staphylococcal complement inhibitor (*scn*) and IgG-binding protein (*sbi*) were each present in over 90% of isolates. This suggests that the majority of circulating strains, regardless of origin, are well-equipped to evade host innate immune responses. These genes, in addition to various resistance mechanisms, may enable the strains to be established in these high-traffic areas and withstand antibiotic selection (Table 4). Our findings extend previous work on the impact of mass gatherings on bacterial populations [18].

**Table 4.** Regional prevalence in % and number of isolates per region and total count for important virulence factors for *S. aureus*

Gene	Role	Jeddah west (*N*=275)	Riyadh central (*N*=158)	Madina west (*N*=102)	Hail north (*N*=64)	Makkah west (*N*=36)	Alhassa east (*N*=33)	Jazan south (*N*=18)	Overall (*N*=686)
PVL	Pore-forming cytotoxin (leukocidin)	13.5% (37/275)	19.0% (30/158)	32.4% (33/102)	6.3% (4/64)	13.9% (5/36)	24.2% (8/33)	22.2% (4/18)	17.0% (121/686)
TSST	Superantigen (toxic shock syndrome toxin-1)	8.0% (22/275)	7.0% (11/158)	13.0% (13/102)	26.0% (17/64)	8.0% (3/36)	6.0% (2/33)	0.0% (0/18)	10.0% (68/686)
*sea*	Superantigen (staphylococcal enterotoxin A)	~30% (~83/275)	~30% (~47/158)	~30% (~31/102)	~30% (~19/64)	~30% (~11/36)	~30% (~10/33)	~30% (~5/18)	~30.0% (~206/686)
ACME	Fitness and colonization (arginine catabolic mobile element)	4.0%(11/275)	0.0% (0/158)	5.9% (6/102)	0.0% (0/64)	0.0% (0/36)	0.0% (0/33)	0.0% (0/18)	2.5% (17/686)
*cna*	Adhesin (collagen-binding protein)	4.7% (13/275)	7.6% (12/158)	2.9% (3/102)	6.3% (4/64)	n/a* (1/36)	3.0% (1/33)	0.0% (0/18)	4.9% (34/686)
*clfA*/*clfB*	Adhesins (clumping factors A/B)	>95% (261/275)	>95% (150/158)	>95% (97/102)	>95% (61/64)	>95% (34/36)	>95% (31/33)	>95% (17/18)	>95% (652/686)
*ica*ABCD	Biofilm formation (polysaccharide adhesin)	>90% (248/275)	>90% (142/158)	>90% (92/102)	>90% (58/64)	>90% (32/36)	>90% (30/33)	>90% (16/18)	>90% (617/686)
Immune evasion cluster	Immune evasion (*sak*, *sbi*, *scn*)	>90%(248/275)	>90% (142/158)	>90% (92/102)	>90% (58/64)	>90% (32/36)	>90% (30/33)	>90% (16/18)	>90% (617/686)

This unique demographic pattern and its influence on MRSA evolution and clonal expansion [6] present both challenges and opportunities for public health interventions. Our findings suggest that large travel hub cities may serve as entry points for emerging strains and resistance patterns, potentially allowing early detection of concerning variants before they achieve wider distribution.

Interestingly, a significant increase in the proportion of novel sequence type assignments in Jeddah (9/14) was observed, and this is the main entry point for international travellers to Hajj and Umrah and provides a further indication that pilgrimage is one of the main drivers of drug resistance and diversity in Saudi Arabia. Beyond the overall diversity patterns, specific lineages warrant particular attention. The presence of CC97 in clinical samples indicates successful establishment in clinical facilities. More investigations are needed to understand the genetic basis of this adaptation and its implications for public health, especially as CC97 ranked second in prevalence across the regions. CC97 was detected as MSSA and MRSA carrying SCC*mec*V associated with the *fusC* gene. Newly identified ST8637 in Madina is an indicator of emerging clones in the community. The antimicrobial resistance in CC97 does not indicate higher resistance across regions, except for the resistance to fusidic acid, which is notable [60].

### Limitations of genomic prediction for antimicrobial resistance

These findings highlight the complex relationship between genotype and phenotype in antimicrobial resistance prediction. While genomic prediction tools showed high precision for certain antibiotics (over 0.98 for beta-lactams and fusidic acid), they performed poorly for other drug classes, particularly aminoglycosides. This variable performance reflects both the complexity of resistance mechanisms and the current limitations of prediction methods.

The GWAS analysis revealed both expected and unexpected genetic associations. While we confirmed established resistance determinants such as *mecA_1* and *mecR1* for beta-lactams, and *ermC* and *msr(A)* for macrolide resistance, we also identified several unexpected associations requiring careful interpretation. For instance, fluoroquinolone resistance showed associations with *hsdM* and *entC2*, likely representing indirect relationships rather than causative mechanisms. Their association is linked to being carried on a well-known mobile genetic element, specifically a staphylococcal pathogenicity island (SaPI) and ΦSa3 prophage recombination region [16,61]. Similarly, the association of fusidic acid resistance with transport-related genes (*natA*, *macB* and *dppB*) rather than expected target site mutations suggests potential limitations in our GWAS approach for detecting point mutations and is limited to presence/absence mechanisms.

This discrepancy between genetic markers and phenotypic resistance highlights several challenges. In particular, resistance mechanisms may involve complex genetic interactions that are not captured by current prediction tools. These may include environmental factors and gene expression regulation, which can contribute to resistance phenotypes independently of genetic markers. The convergence of diverse international strains in our study setting may facilitate the exchange of resistance determinants through mobile genetic elements, complicating genotype–phenotype relationships.

Given these limitations, we would suggest maintaining phenotypic testing alongside genomic surveillance for effective clinical decision-making [62]. Such a dual approach is especially crucial in regions experiencing high levels of international travel and strain diversity, where rapid evolution and transmission of resistance mechanisms may occur. Future improvements in prediction accuracy will likely require integration of multiple data types, including transcriptomics and proteomics, to better capture the complex determinants of antimicrobial resistance. Moreover, this integrated data can provide an important insight into the newly emerging phage therapy design [63]

### Data availability and reproducibility

A key contribution of this study is the comprehensive implementation of FAIR principles [19] in data sharing. We structured our metadata using the RDF [44], creating a semantically rich representation of both sequence data and phenotypic measurements. Our data model (Fig. S3) incorporates standard terminologies and ontologies, including the National Center for Biotechnology Information (NCBI) Taxonomy [64] for species annotation, the ChEBI ontology [65] for antimicrobial agents, the NCI Thesaurus [66] and the GENEPIO ontology [67] for health status descriptions and the unit ontology [68] for standardized units, enhancing interoperability with existing datasets.

This structured approach to data sharing serves multiple purposes. First, it enables direct integration with other surveillance datasets through standardized terminology and relationships. The use of controlled vocabularies for key metadata elements – such as specimen sources, host characteristics and antimicrobial susceptibility measurements – facilitates automated data integration and comparative analyses across different studies and geographical regions.

Second, the dataset reported here is particularly valuable for developing and validating machine learning approaches for antimicrobial resistance prediction. By providing paired genomic and phenotypic data in a standardized format, along with detailed host and environmental metadata, we enable researchers to develop prediction models that can account for contextual factors beyond purely genetic determinants and may be more adapted to the complex demographics found among pilgrims. The comprehensive antimicrobial susceptibility data, including both MIC values and categorical interpretations, further provides rich training and validation data for such models.

The complete computational reproducibility of our analysis, implemented through a workflow in the CWL [42], ensures that other researchers can not only access our data but also reproduce and build upon our analytical methods. By making our workflows, source code and execution environments publicly available, we facilitate easier adaptation of our methods to new datasets and research questions.

Implementation of FAIR principles extends beyond technical accessibility to practical reusability. The standardized metadata format allows researchers to integrate our findings with their local surveillance data, potentially revealing new patterns of MRSA transmission and evolution. Furthermore, our dataset can serve as a baseline for tracking changes in MRSA populations, particularly in the context of mass gatherings and international travel, where standardized data collection and sharing are crucial for effective surveillance.

### Future work

Our findings, as reported, point to several key directions for future surveillance strategies, particularly focusing on early detection at pilgrimage entry points. The implementation of systematic sampling could be strategically enhanced by targeting major travel hubs. Specifically, wastewater surveillance at airports serving pilgrimage routes, particularly the international airports in Jeddah and Madina, could provide early warning signals of emerging strains. This approach is important because our results show that pilgrimage cities harbour significantly higher resistance rates and novel sequence types, thereby indicating that they are the locations where new resistant variants first appear in the country. Aircraft wastewater sampling from pilgrimage flights, which has proven effective for Coronavirus Disease 2019 (COVID-19) surveillance [69], could be adapted for MRSA monitoring, offering insights into strain importation patterns before pilgrims arrive at their destinations. Further, microevolution studies can be performed using our dataset and tools similar to ClonalFrameML, which explicitly models both mutation and recombination events to infer evolutionary relationships more accurately. This approach will allow us to investigate potential transmission clusters across cities [70].

Such a targeted approach could be complemented by environmental sampling at key congregation points along pilgrimage routes. Strategic sampling points could include ablution facilities in major mosques and shared sanitation facilities in pilgrim accommodations, providing a comprehensive picture of strain circulation during mass gatherings. Integration of these environmental samples with clinical surveillance would create a more complete understanding of MRSA transmission dynamics during mass gatherings.

The application of long-read sequencing technologies to these samples could resolve complex genetic elements that are challenging to characterize with short-read data alone. This would be particularly valuable for understanding the structure of SCC*mec* cassettes and other mobile genetic elements that may be exchanged during mass gatherings.

Finally, establishing a real-time surveillance system that combines this strategic sampling with rapid genomic and phenotypic analysis would enable faster response to emerging resistant strains. Such a system could serve as a model for monitoring other pathogens in similar mass gathering settings, contributing to global pathogen surveillance efforts and early warning systems for emerging antimicrobial resistance.

## Conclusion

This study reveals that mass gathering cities in Saudi Arabia serve as hotspots for MRSA diversity and antimicrobial resistance, with pilgrimage-associated locations showing significantly higher resistance rates (33.9% vs. 27.6%) and harbouring the majority of novel sequence types. While pilgrimage cities exemplify the intersection of global human mobility with pathogen evolution, the distinct patterns we observed in non-pilgrimage regions highlight the importance of local healthcare practices and potential livestock–human transmission routes in shaping MRSA populations. This comprehensive view, spanning both high-traffic pilgrimage sites and regional healthcare settings, provides evidence for a framework to understand pathogen dynamics in complex environments where international, community and agricultural factors converge. Through standardized surveillance approaches and data sharing, our work establishes a foundation for monitoring antimicrobial resistance in the context of both global human mobility and regional One Health challenges, with implications for public health strategies across the Middle East and beyond.

## Supplementary material

10.1099/mgen.0.001540 Uncited Supplementary Material 1.

## Abbreviations

CCs: clonal complexes
CLSI: Clinical and Laboratory Standards Institute
COVID-19: Coronavirus Disease 2019
CWL: common workflow language
FAIR: findability, accessibility, interoperability and reusability
GWAS: genome-wide association study
MIC: minimum inhibitory concentration
MLST: multilocus sequence types
MRSA: methicillin-resistant *Staphylococcus aureus*
MSSA: methicillin-susceptible *Staphylococcus aureus*
NCBI: National Center for Biotechnology Information
P: precision
PVL: Panton–Valentine leukocidin
R: recall
RDF: Resource Description Framework
SCC*mec*: staphylococcal chromosomal cassette *mec*
STs: sequence types
TSST: toxic shock syndrome toxin
